# The CXCL12γ Chemokine Displays Unprecedented Structural and Functional Properties that Make It a Paradigm of Chemoattractant Proteins

**DOI:** 10.1371/journal.pone.0002543

**Published:** 2008-07-02

**Authors:** Patricia Rueda, Karl Balabanian, Bernard Lagane, Isabelle Staropoli, Ken Chow, Angelique Levoye, Cedric Laguri, Rabia Sadir, Thierry Delaunay, Elena Izquierdo, Jose Luis Pablos, Elena Lendinez, Antonio Caruz, Diego Franco, Françoise Baleux, Hugues Lortat-Jacob, Fernando Arenzana-Seisdedos

**Affiliations:** 1 Departamento de Biología Experimental, Universidad de Jaén, Jaén, Spain; 2 Viral Pathogenesis laboratory, Institut Pasteur, Paris, France; 3 INSERM U819, Paris, France; 4 Institute for Structural Biology, Gagophile laboratory UMR 5075 CNRS-CEA-UJF, Grenoble, France; 5 INRA, Villenave d'Ornon, France; 6 Servicio de Reumatología y Unidad de Investigación, Hospital 12 de Octubre, Madrid, Spain; 7 Unité de Chimie Organique, Institut Pasteur, Paris, France; Oregon Health & Science University, United States of America

## Abstract

The CXCL12γ chemokine arises by alternative splicing from *Cxcl12*, an essential gene during development. This protein binds CXCR4 and displays an exceptional degree of conservation (99%) in mammals. CXCL12γ is formed by a protein core shared by all CXCL12 isoforms, extended by a highly cationic carboxy-terminal (C-ter) domain that encompass four overlapped BBXB heparan sulfate (HS)-binding motifs. We hypothesize that this unusual domain could critically determine the biological properties of CXCL12γ through its interaction to, and regulation by extracellular glycosaminoglycans (GAG) and HS in particular. By both RT-PCR and immunohistochemistry, we mapped the localization of CXCL12γ both in mouse and human tissues, where it showed discrete differential expression. As an unprecedented feature among chemokines, the secreted CXCL12γ strongly interacted with cell membrane GAG, thus remaining mostly adsorbed on the plasmatic membrane upon secretion. Affinity chromatography and surface plasmon resonance allowed us to determine for CXCL12γ one of the higher affinity for HS (K_d_ = 0.9 nM) ever reported for a protein. This property relies in the presence of four canonical HS-binding sites located at the C-ter domain but requires the collaboration of a HS-binding site located in the core of the protein. Interestingly, and despite reduced agonist potency on CXCR4, the sustained binding of CXCL12γ to HS enabled it to promote *in vivo* intraperitoneal leukocyte accumulation and angiogenesis in matrigel plugs with much higher efficiency than CXCL12α. In good agreement, mutant CXCL12γ chemokines selectively devoid of HS-binding capacity failed to promote *in vivo* significant cell recruitment. We conclude that CXCL12γ features unique structural and functional properties among chemokines which rely on the presence of a distinctive C-ter domain. The unsurpassed capacity to bind to HS on the extracellular matrix would make CXCL12γ the paradigm of haptotactic proteins, which regulate essential homeostatic functions by promoting directional migration and selective tissue homing of cells.

## Introduction

The CXC chemokine, stromal cell-derived factor 1/CXCL12 [1] is a constitutive and broadly expressed chemokine that exerts its functions through the G-protein coupled receptor (GPCR) CXCR4 [2]. Recently, a novel receptor for CXCL12, RDC-1/CXCR7, has been identified [3]–[5]. Mouse and human CXCL12α, the major CXCL12 isoform, differ by a single, homologous substitution (Val18 to Ile18) [1], [6] and each protein owns the capacity to bind and activate the orthologue CXCR4 receptor. The exceptional conservation of both CXCR4 and CXCL12 structure and function in mammalians announces the essential roles played by this singular couple. CXCL12 is unique among the family of chemokines as it plays non-redundant roles during embryo life in the development of both cardiovascular [7] and central nervous system [8], [9], hematopoiesis [10] and colonization of the gonads by primordial germ cells [11]. In the post-natal life, CXCL12 is involved in trans-endothelial migration of leukocytes [12]–[15] and regulates critically both the homing and egress of CD34+CXCR4+progenitor cells from the bone marrow, and their migration into peripheral tissues [16]. CXCL12 also plays a prominent role in physiopathological processes such as inflammation [17], angiogenesis and wound healing [18], [19]. Moreover, CXCL12 is a critical factor for growth, survival and metastatic dissemination of a number of tumors [20].

The engagement of CXCR4 by CXCL12 triggers the activation of heterotrimeric Gαβγ-proteins, which ultimately promote the directional migration of cells towards a concentration gradient of ligand that defines the haptotactic function of chemokines. *In vivo*, chemokines are believed to form gradient concentrations by binding to glycosaminoglycans (GAG), the glycanic moieties of proteoglycans, and in particular to heparan sulfate (HS). Electrostatic contacts between the negatively charged HS and basic residues exposed at the surface of chemokines, along with structural features of the oligosaccharide, determine both the affinity and the specificity of the molecular interactions that are supposed to modulate the *in vivo* biological activity of chemokines complexed to proteoglycans [21]–[24].

The study of the well characterized CXCL12α isoform provided most of the knowledge of CXCL12 biological properties including interaction with GAG, which is essentially accounted for by a canonical BBXB (B for basic amino-acids, X any other amino-acid) HS-binding motif, located in the first β-strand of the protein [25]. In contrast, the novel CXCL12γ isoform remains largely unexplored regarding protein expression and biological function. CXCL12γ is formed by a core domain encompassing the 68 amino-acids of the major CXCL12α isoform shared with all CXCL12 proteins, which is extended by a carboxy-terminal (C-ter) domain. This region, highly-enriched in basic amino-acids, encodes four overlapped HS-binding motifs and shows identical sequence in human, rat and mouse species [6], [26], [27]. This positively charged domain enables CXCL12γ with an amazing capacity to interact with GAG [28]. We speculated that this property might be determinant in defining the *in vivo* capacity of this peculiar chemokine to promote both migration and homing of cells in tissues. In this work we characterized CXCL12γ tissue expression and the capacity of this isoform to interact with CXCR4 and promote cell migration *in vitro*. Moreover, we investigated the interaction of CXCL12γ with GAG both *in vitro* and on intact cells. Finally, we assessed the functionality of this novel isoform *in vivo*. Our findings indicate that CXCL12γ displays a sustained binding on GAG and exhibits a prolonged chemokine activity *in vivo* that makes it a paradigm among haptotactic proteins. The intacteness of the BBXB sites in the distinctive CXCL12γ C-ter domain critically determine the biological activity of the chemokine.

## Results

### Tissue distribution of Cxcl12γ products

**Figure 1:**
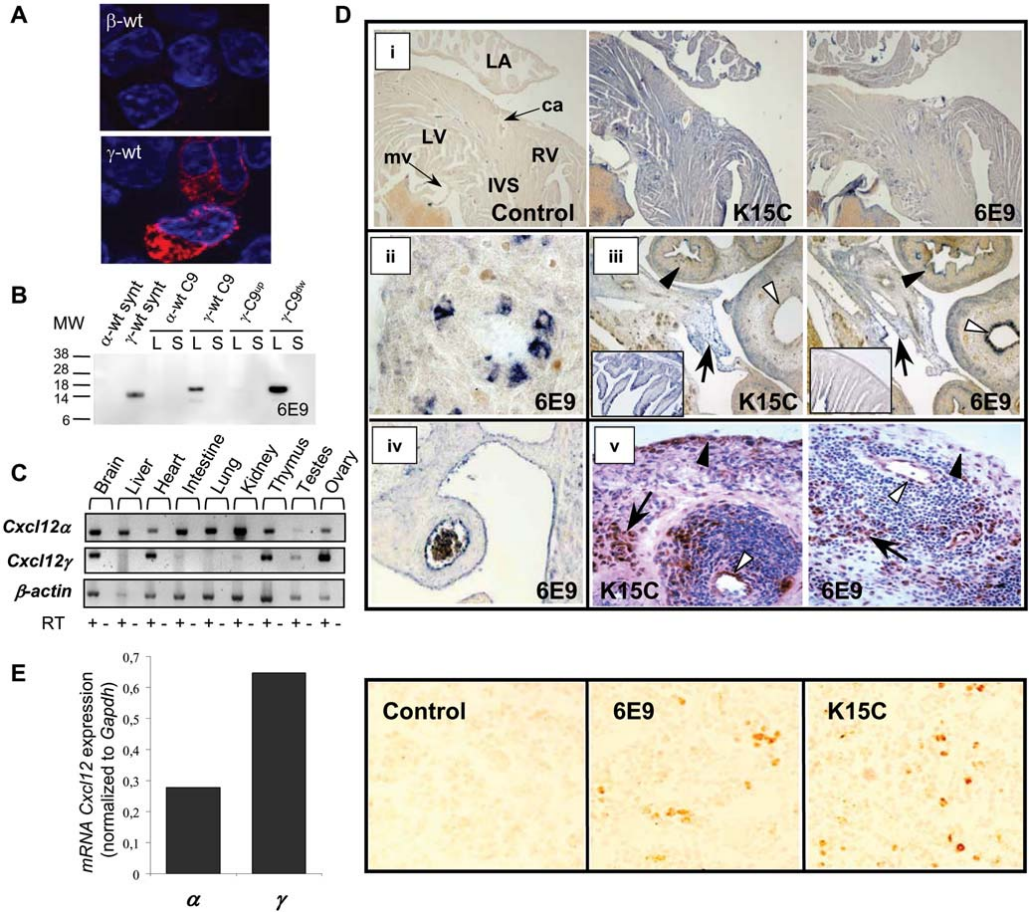
Tissue expression of γ-wt in human and mouse. (A) Specific immunofluorescent detection of γ-wt. HEK-293T cells were transfected either with β-wt- (upper panel) or γ-wt- (lower panel) expressing pCDNA3.1 plasmids, treated with Brefeldin A, permeabilised with saponin and labelled with the 6E9 mAb and a Texas Red anti-mouse IgG. The nuclei of cells were counterstained with DAPI. Images are representative of six independent determinations. Original magnification ×63. (B) Mutagenesis of K78/K80 in γ-wt C9 (γ-C9^up^) prevents the specific recognition of the γ-wt chemokine by the 6E9 mAb. Western blot analysis of chemically synthesized α-wt and γ-wt (synt) and α-wt C9, γ-wt C9, γ-C9^up^ and γ-C9^dw^ C9-tagged chemokines expressed from SFV-vectors in BHK cells. Cell lysates (L) or culture supernatants (S) were separated by SDS-PAGE and probed with 6E9 mAb and a HRP-sheep anti-mouse Ig secondary antibody. MW, molecular weight in KDalton. Results are representative of two independent determinations. (C) Expression of *Cxcl12α* and *Cxcl12γ* mRNAs by RT-PCR in different adult mouse tissues. β-actin was used as loading control. RT +/− denotes presence or absence of RT enzyme. Data are representative of three independent determinations. (D) Detection of CXCL12 isoforms either with K15C mAb or anti-γ-wt 6E9 mAb in mouse and human tissues. (i) Mouse adult heart. LA, left auricle; LV, left ventricle; RV, right ventricle; IVS, interventricular septum; ca, carotid artery; mv, mitral valve. (ii) Detail of a lung bronchiol (mouse E16.5 embryo). (iii) Mouse E16.5 embryo intestin and bladder. White arrowheads, bladder epithelium; black arrowheads, large intestine; arrows, peritoneum. In inset, details of intestinal mucosa labeling. (iv) Large abdominal vessel (mouse E16.5 embryo). (v) Human inflammatory synovial tissue (rheumatoid arthritis). White arrowheads, blood vessel; black arrowheads, lining synoviocytes; arrows, fibroblasts. Control: secondary antibody. Original magnifications ×4 (i,iii inset), ×10 (iii), ×20 (iv), ×40 (ii) and ×400 (v). (E). CXCL12 expression in the mouse bone marrow. Left panel, expression of *Cxcl12α* (*α*) and *Cxcl12γ* (*γ*) mRNAs determined by quantitative real time-PCR and normalized to *Gapdh* expression. Results are representative from three independent determinations for each PCR reaction. Right panel, detection of CXCL12 isoforms by use of either K15C or anti-γ-wt 6E9 mAb. Control: secondary antibody. Original magnification (×40).

The *Cxcl12γ* isoform cDNA was obtained from BALB/c mouse brain mRNA. The isolated cDNA nucleotide sequence was identical to the previously reported murine *Cxcl12γ* isoform (GenBank NCBI accession number NM_001012477) that encodes the CXCL12γ protein (thereafter called γ-wt for the recombinant and chemically synthesized proteins, GenPept NCBI accession number NP_001012495). The expression of the γ-wt mRNA and protein in embryo and adult mouse tissues and in human adult tissues was investigated by RT-PCR and immunohistochemistry (Figure 1C–E) using a novel monoclonal antibody (mAb) (6E9) that recognizes selectively a γ-wt C-ter epitope encompassing the sequence K78/K80 (Figure 1A–B). The γ-wt protein expression was compared to these of other isoforms detected by the well characterized K15C mAb, which recognizes an amino-terminal (N-ter) -encoded epitope shared by all the CXCL12 isoforms [29], [30].

In adult mice, the *Cxcl12γ* mRNA was poorly expressed in liver, intestine and kidney, contrasting with the abundant expression of *Cxcl12α* mRNA (Figure 1C). Regarding the protein (Figure 1D), γ-wt was undetectable in bladder muscular and mucosa layers, while in the intestinal tract, a faint and discontinuous immunostaining was restricted to the mucosa and excludes the muscular layer (Figure 1Diii). *Cxcl12γ* mRNA was abundant in brain, heart (Figure 1C) and bone marrow, where it was expressed as a predominant isoform akin to *Cxcl12α* as quantified by real time PCR (Figure 1E). γ-wt protein was detected in cardiac muscle, valves and large vessels (Figure 1Di). In lungs, *Cxcl12γ* mRNA expression was barely detected in the adult (Figure 1C). Interestingly, a detailed analysis of γ-wt expression in mouse embryos showed that while the protein was virtually absent from trachea and large bronchia, it accumulated in the bronchioli (Figure 1Dii). The γ-wt protein was consistently detected in mesothelial tissues such as peritoneum (Figure 1Diii) and pleura (data not shown). Of note, γ-wt was detected in endothelia of large and small vessels both in human and mouse (Figure 1Div-v), and in fibroblasts either of human skin (data not shown) or synovial inflammatory tissue (rheumatoid arthritis; Figure 1Dv).

### γ-wt binds to immobilized and cell surface HS with high affinity

**Figure 2:**
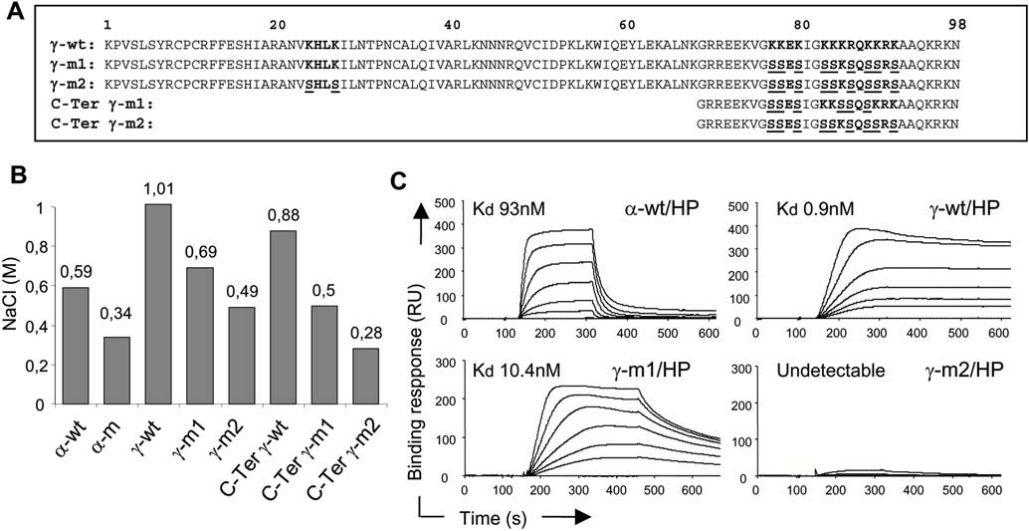
Immobilized GAG-binding activity of γ-wt. (A) Sequence alignment of CXCL12γ wt (γ-wt), mutated derivatives chemokines (γ-m1 and γ-m2) and mutant CXCL12γ C-ter peptides (C-Ter γ-m1 and C-Ter γ-m2). In bold, identified and putative HS-binding motifs; underlined, substituted amino acids. (B) Binding to HP-affinity columns of chemically synthesized wt (α-wt, γ-wt) or mutant (α-m, γ-m1, γ-m2) chemokines or peptides encompassing amino acids 69 to 98 of CXCL12γ wt (C-ter γ-wt) or their mutated derivatives (C-ter γ-m1, C-ter γ-m2). Proteins were applied to heparin Hitrap-columns and eluted with a 0.15 to 1 M NaCl gradient. Values correspond to the NaCl molar (M) concentration required for elution from the heparin column and represent three independent experiments. (C) Binding of α-wt, γ-wt, γ-m1 or γ-m2 to on chip-immobilized heparin (HP). Chemokines were injected over HP activated surface for 5 min, after which running buffer was injected, and the response in relative units (RU) was recorded as a function of time. Each set of sensorgrams was obtained with α-wt at (from top to bottom) 200 to 0 nM or γ-wt, γ-m1 and γ-m2 at 25 to 0 nM. Results are representative of three independent determinations.

Previously it has been shown that the CXCL12α protein (α-wt) binds with high affinity to HS [31] both *in vitro* and in intact cells through specific interaction with the canonical HS-binding motif (K24H25L26K27) located in the core of the protein shared by all the CXCL12 isoforms. Mutation of this motif (K24S/K27S) fully prevents binding to HS without affecting neither the overall structure nor the capacity of the mutant chemokine (α-m) to bind and activate CXCR4 [31]. The specific C-ter domain of the γ-wt isoform presents a marked basic character, with a 60% of the residues being positively charged and clustered in 4 overlapped HS-binding sites. This prompted us to investigate the γ-wt/GAG interactions both *in vitro* and on intact cells. Analysis performed with chemically synthesized chemokines, showed that γ-wt isoform required 1.01 M NaCl to be eluted from a heparin (HP)-affinity column (Figure 2B) as compared to 0.59 M required for elution of α-wt. Chemically synthesized γ-wt C-ter peptide encompassing amino-acids 69 to 98 of the corresponding γ-wt protein required 0.88 M NaCl to be eluted, indicating that this domain interacts with HP *per se* with high affinity and might contributes to the strong interaction with HP displayed by γ-wt. In good agreement, neutralization of positively charged amino-acids by mutation of the C-ter BBXB motifs either in the γ-wt (γ-m1, Figure 2A) or the isolated C-ter peptides (C-ter γ-m1 and C-ter γ-m2, Figure 2A), reduced drastically the ionic force (0.69, 0.5 and 0.28 M NaCl, respectively, Figure 2B) required for their elution from the HP-affinity column.

Surface plasmon resonance (SPR) experiments (Figure 2C) confirmed that γ-wt interacts with HP with unprecedented high affinity (K_d_ = 0.9 nM). Furthermore, they showed that the interaction with the oligosaccharide was severely impaired in the mutant γ-m1 (K_d_ = 10.4 nM), thus proving the important contribution of the C-ter domain BBXB sites to the binding on HP. Both HP-affinity chromatography and SPR experiments (Figure 2B–C) proved that the γ-m2 mutant (Figure 2A), which lacks all functional BBXB motifs, was virtually devoid of the capacity to interact with HP.

**Figure 3:**
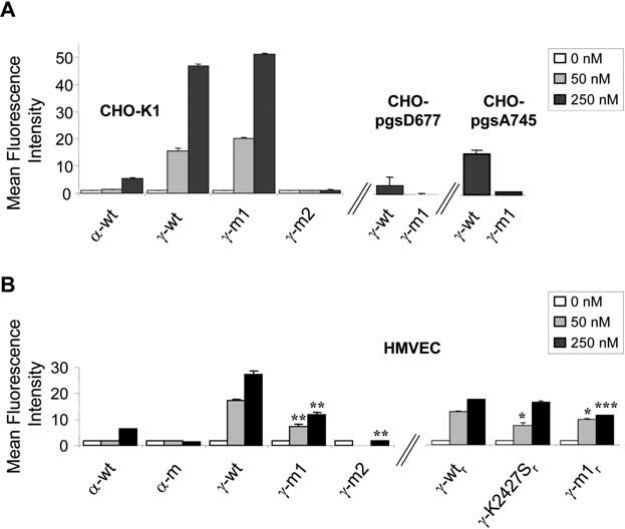
Cell surface GAG-binding activity of α-wt and γ-wt. (A) Parental (K1) or GAG-mutant (pgsD677, pgsA745) CHO cells were incubated with the indicated concentration of wt (α-wt, γ-wt) or mutant (γ-m1, γ-m2) chemokines for 60 min at 4°C, and after extensive washing to remove free chemokine, were labelled with K15C mAb and a PE-goat anti-mouse Ig secondary antibody. Fixed cells were analyzed by flow cytometry. Values represent the mean fluorescence intensity±SD of three independent experiments performed in triplicate (B) Primary human-microvascular endothelial cells (HMVEC) were incubated with the indicated concentration of chemically synthesized (α-wt, α-m, γ-wt, γ-m1, γ-m2; left) or recombinant (γ-wt_r_, γ-K2427S_r_, γ-m1_r_, right) chemokines and treated as in (A). Values represent the mean fluorescence intensity±SD of two independent experiments performed in triplicate. *p<0.05, **p<0.01, ***p<0.005 as compared to the binding obtained for the corresponding concentration of γ-wt (for γ-m1 and γ-m2) or γ-wt_r_ (for γ-k2427S_r_ and γ-m1_r_) chemokines.

Recognition of CXCL12 proteins by the K15C mAb is not masked by their interaction with GAG [23]. Using this mAb, we observed that the adsorption on the CXCR4 negative CHO-K1 cells was greatly increased for γ-wt as compared to α-wt (Figure 3A). Of note, and of particular biological relevance, we found that γ-wt also binds onto primary, human-microvascular endothelial cells (HMVEC) with the highest efficiency as compared to α-wt (Figure 3B). It is interesting to note that while the γ-m1 mutant protein retained the capacity to bind on CHO-K1 parental cells, this capacity was notably decreased in HMVEC, a divergence that could be accounted for by differences in the amount and nature of negatively charged structures that contribute to CXCL12 binding in both cell types. Interestingly, a recombinant CXCL12γ derivative carrying K24S and K27S substitutions (γ-K2427S_r_) that invalidate the HS-binding consensus site located in the core of the protein [25] (Figure 3B) also exhibits a reduced capacity to bind on HMVEC cells as compared to the corresponding γ-wt_r_ protein. In keeping with these results, the γ-m2 was virtually devoid of any binding capacity on both cell types. The specificity of CXCL12γ binding to GAG was assessed in mutant CHO-pgsD677 cells, derived from CHO-K1 cells, which lack both *N*-acetylglucosaminyltransferase and glucuronyltransferase activities and are deficient for HS synthesis, the binding of γ-m1 became undetectable, whereas a residual signal was still detectable for γ-wt (Figure 3A), and at a similar extent for the γ-K2427S_r_ (data not shown). Comparable phenomena were observed in CHO-pgsA745 cells, which lack any GAG synthesis due to a xylose-transferase mutation. The residual binding of γ-wt (or γ-K2427S_r_) observed at high concentrations of the chemokine (250 nM), in the absence of any synthesized GAG, can be accounted for by the interaction of the C-ter domain with other negatively charged structures, like the abundant sulphate glycosphingolipids (sulphatides) that have been previously shown to interact at high concentrations with α-wt [32]. The enzymatic degradation of HS in CHO-K1 cells either by heparinase or heparitinase I confirmed the apparent selectiveness of the HS/γ-wt interaction at the cell surface, whereas degradation of chondroitin sulfate had no effect (Figure S1).

Collectively these results demonstrate the hypothesis that both the protein core and C-ter HS-binding sites collaborate to provide CXCL12γ with the characteristic and unchallenged, high affinity binding for GAG, and prove the validity of this assumption in cell models of biological relevance.

### Neosynthesized γ-wt shows an unusual pattern of cell secretion and accumulation

**Figure 4:**
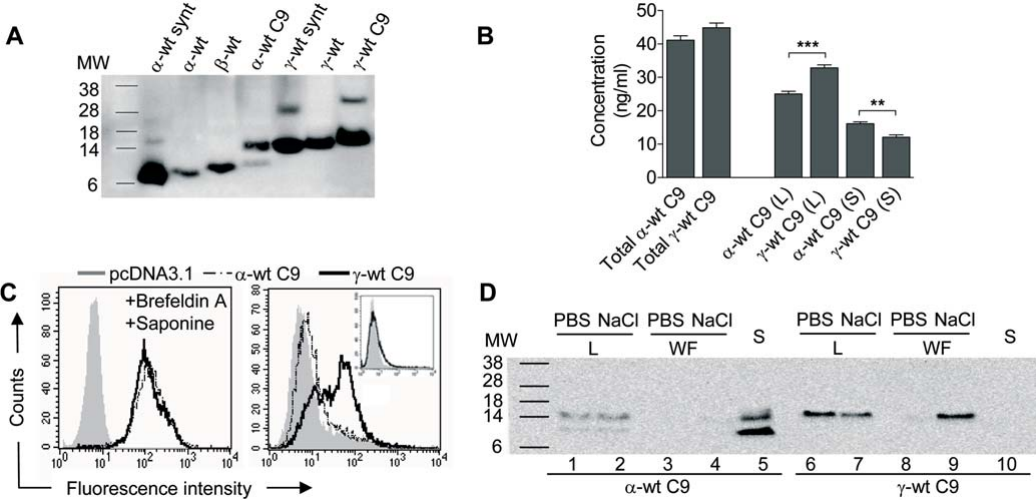
Electrophoretic mobility and secretion pattern of γ-wt and α-wt chemokines. (A) For detection of CXCL12 proteins by immunoblot, SFV-infected BHK cell lysates were separated by SDS-PAGE and probed with the K15C mAb and a HRP-sheep anti-mouse Ig secondary antibody. Abbreviations like in Fig. 1B. Formation of dimeric forms are observed for α-wt synt, α-wt C9, γ-wt synt and γ-wt C9. Results are representative of three independent determinations. (B) ELISA quantification of α-wt C9 and γ-wt C9 expressed by HEK-293T cells transfected with the corresponding pcDNA3.1 expression vector. Total protein or chemokine accumulated in the cell supernatant (S) or in the cell lysates (L) were determined. The values (ng/ml) are mean±SD from triplicate measurements. **p<0.005, ***p<0.0005. (C) The secreted γ-wt C9 protein revealed by the anti-C9 1D4 mAb accumulates massively at the cell surface of HEK-293T cells treated like in (B), and permeabilised (left panel) or not (right panel) with saponin. In inset, cell surface CXCR4 expression of HEK-293T cells. Results are representative of four independent determinations. (D) The γ-wt C9 chemokine is released from intact cells upon exposure to strong ionic force. BHK cells were infected with SFV-infectious particles driving the expression either of α-wt C9 or γ-wt C9. Thereafter, the proteins were detected by western blot analysis using the anti-C9 1D4 mAb in the cell culture supernatant (S), the wash fluid (WF) or the cell lysate (L), upon a 5 min exposure of cells either to PBS or hypertonic NaCl 1 M (NaCl). Results are representative of four independent determinations.

For ease of detection, the sequence coding for a 9 amino acid C-ter (C9-tag) peptide from bovine rhodopsin that has been satisfactorily used for tagging a large number of unrelated proteins, was added in frame at the 3′ end of the open reading frames (ORF) of the *Cxcl12α*- and *Cxcl12γ*-encoding constructs, giving rise to the α-wt C9 and γ-wt C9 proteins, respectively. Chemokines were expressed in BHK cells by infection with Semliki forest virus (SFV) particles expressing the corresponding C9-tagged isoform. We observed that, upon expression, the γ-wt C9 protein was hardly detectable by western blot analysis in the BHK cell culture supernatants (Figures 1B and 4D). This finding prompted us to investigate the fate of this protein and to compare it to that of α-wt C9 which was engineered and expressed under identical experimental conditions (Figure 4A). Quantification in an ELISA assay showed that similar amounts of γ-wt C9 and α-wt C9 were produced from expressing cells (Figure 4B). Moreover, quantification of either cell-associated (cell lysate) or free chemokines (supernatant) reveled that a larger fraction of γ-wt C9 remained associated to cells as compared to α-wt C9. To further investigate the distribution of the chemokine fraction associated to cells, we performed labelling of α-wt C9- and γ-wt C9-expressing cells using the anti-C9 1D4 mAb (Figure 4C). Interestingly, γ-wt C9 markedly amassed at the cell surface in contrast to α-wt C9 (Figure 4C right panel), while in the presence of Brefeldin A, similar amounts of each chemokine accumulated in intracellular stores (Figure 4C left panel). Enzymatic exposure to heparitinase I reduced the intensity of the signal for both chemokines (data not shown), which is in full agreement with the previous SPR data showing that γ-wt binds with exceptionally high affinity to on-chip immobilized HS (Figure 2C) and the results obtained from the enzymatic treatment of CHO-K1 cells (Figure S1). These findings led us to postulate that given the cationic nature of the C-ter of γ-wt and its high affinity for GAG, electrostatic forces enable this chemokine to bind tightly to negatively charged structures at the cell surface. This assumption was tested by shortly exposing SFV-infected BHK cells expressing either γ-wt C9 or α-wt C9 to isotonic PBS or 1 M NaCl solution, in order to disrupt the electrostatic interactions and eventually promote release of the chemokine into the fluid (Figure 4D). Cell viability of NaCl-treated cells was not altered as compared to this of control cells (data not shown) when assessed by blue-trypan dye exclusion. While α-wt C9-expressing cells did not release detectable amounts of this isoform in the wash fluid (Figure 4D lanes 3–4), a significant amount of γ-wt C9 was released upon short exposure of cells to 1 M NaCl solution (Figure 4D lane 9). These findings were reproduced in other cell types (HEK 293T) and with different expression vectors (pcDNA3.1).

### γ-wt displays reduced agonist potency on CXCR4 activation as compared to α-wt

**Figure 5:**
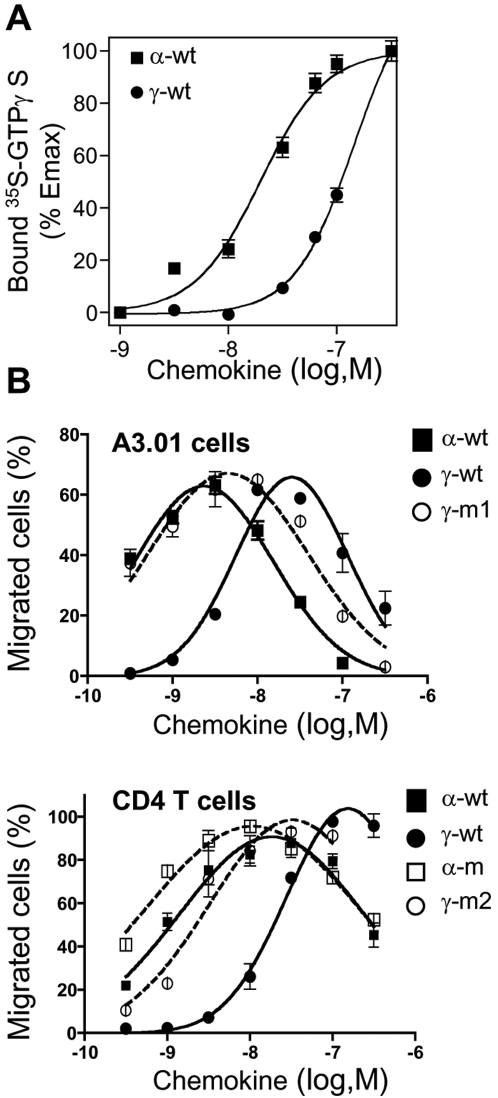
Cell signalling through CXCR4 induced either by α-wt, γ-wt or derivative chemokines. (A) CXCL12-induced [^35^S]GTPγS binding to membranes from lymphoblastoid A3.01 T cells. Membranes were incubated in assay buffer containing 0.1 nM [^35^S]GTPγS and the indicated concentrations of the corresponding chemokine. Data represents the percentage (mean±SD) of the maximal [^35^S]GTPγS binding obtained (100%), and are representative of three independent experiments. (B) Dose-dependent CXCL12-induced chemotaxis of A3.01 cells (upper panel) or primary CD4+ T lymphocytes (lower panel). Results (mean±SD) are from two independent experiments and are expressed as percentage of input cells that migrated to the lower chamber.

The pharmacological properties of γ-wt regarding its interaction with CXCR4 were investigated on transformed A3.01 T cells and primary unstimulated CD4+ T lymphocytes (Figure 5). Both lymphoid cell types lack detectable levels of HS as assessed by immunostaining with the specific 10E4 anti-HS mAb (data not shown) and permits the strict analysis of CXCL12/CXCR4 interaction *per se*. The capacity of γ-wt to set in motion CXCR4-dependent activation cell pathways was first assessed by measuring the amount of the non-hydrolysable [S^35^]-GTPγ associated to activated Gα subunits, the earliest cell-signal event induced by GPCR agonists. We observed that γ-wt was less potent than α-wt to activate CXCR4 (Figure 5A), which is in full agreement with the reduced binding affinity (one order of magnitude) shown by γ-wt for CXCR4 as compared to α-wt [28]. When concentration of γ-wt was raised and the occupancy of CXCR4 was enhanced, G protein activation increased to levels comparable to those measured with α-wt. However, presumably due to the sustained reduced potency of γ-wt to induce GTPγS binding, we were unable ro reach saturation for γ-wt, thus precluding determination of E_max_ and EC_50_ values for this chemokine in ths assay. For α-wt isoform, we deduced an EC_50_ value equal to 19.65 nM. CXCL12α has been shown to bind to- and activates CXCR7 [3], [4], [33]. We thus analysed the ability of γ-wt to compete with a C-ter biotinylated CXCL12α chemokine for binding to CXCR7. As shown in Figure S2, α-wt and γ-wt isoforms similarly bound to CXCR7 (IC_50_ = 6.56 nM and 10.37 nM for α-wt and γ-wt, respectively).

We next investigated the capacity of γ-wt to promote CXCR4-mediated lymphocyte migration, the hallmark of chemokine-promoted responses (Figure 5B). Addition of γ-wt to A3.01 cells (upper panel) or primary CD4+ T lymphocytes (lower panel) confirmed, in agreement with previous findings [33], the reduced potency of γ-wt as compared to α-wt regarding chemotactic activity. Addition of the specific CXCR4 antagonist AMD3100 resulted in the blockade of both G-protein coupling and cell migration, thus proving the specificity of CXCR4/γ-wt interactions (Figure S3).

Invalidation of the C-ter BBXB motifs in γ-wt (γ-m1 and γ-m2) restored the potency of the chemokine to the levels showed by α-wt and α-m, two chemokines that have been previously shown to bind to and activate CXCR4 similarly [31]. Comparable results were found regarding GTPγS binding, as the γ-m1 mutant proved to be consistently a better agonist than γ-wt, displaying a similar potency than this observed for α-wt (data not shown). Additionally, these findings indicated that the mutations introduced in the C-ter domain did not affect the overall structure of the chemokine. The biological relevance of these results was further confirmed in human blood leukocytes activated with phytohemagglutinin and IL-2, a process known to enhance GAG expression on primary cells [34] (Figure S4).

### In vivo biological activity of γ-wt

**Figure 6:**
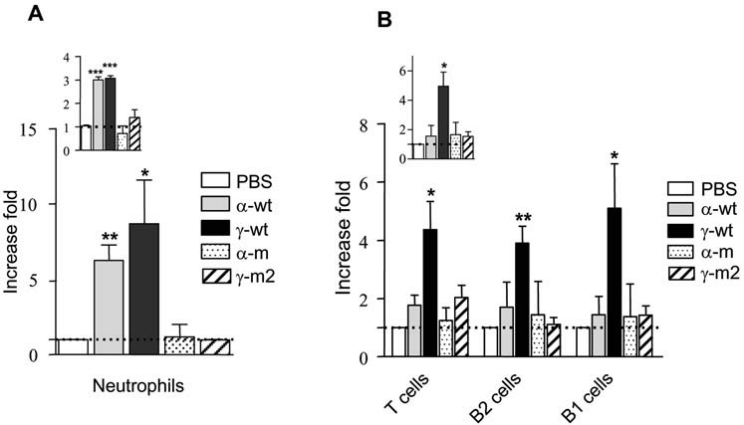
Intraperitoneal recruitment of leukocytes induced by α-wt and γ-wt. BALB/c mice were intraperitoneally injected with identical volumes (300 µl) of either PBS (control) or a 30 nM solution of each chemokine. Cells that accumulated into the peritoneal cavity were recovered after 6 hr (A) or 15 hr (B) of treatment. Leukocyte subpopulations were characterized by flow cytometry using specific cell markers. Cell influx was calculated as the x-fold increase over values obtained in PBS-treated mice. PBS reference values were arbitrary set to one (dotted lines). In inset, total number of recovered peritoneal cells. Results are mean±SD of three independent experiments. *p<0.05, **p<0.01, ***p<0.005 as compared to PBS-treated mice.

The singular structural and functional features that distinguish γ-wt from α-wt prompted us to compare their respective capacities to promote haptotactic attraction of cells *in vivo* using chemokine concentrations in the range of these that consistently induce chemotaxis *in vitro*. To this purpose, we first evaluated the migration of leukocytes into the peritoneal cavity of BALB/c mice following administration of an endotoxin-free, 30 nM solution, of γ-wt or α-wt at 6 hours (hr) (Figure 6A) or 15 hr (Figure 6B) post-injection. After 6 hr of treatment, both α-wt and γ-wt induced a significant and equivalent increase of the absolute number of cells (fold increase 2.99±0.18 and 3±0.18, for α-wt and γ-wt respectively, as compared to control PBS injected animals), that was accounted for by the recruitment of myeloid cells, including both neutrophils and macrophages (Gr-1+CD11b+CD19-, 6.27±1.45 for α-wt and 8.72±4.95 for γ-wt). The situation was radically different at 15 hr post-injection as solely γ-wt promoted a sustained accumulation of leukocytes (fold increase 4.96±1.35 as compared to PBS-injected animals). At this time point, cell increase was basically accounted for by T lymphocytes (CD3+, 4.38±1.65) and B lymphocytes corresponding to B1 (CD19+CD11b+, 5.09±2.16) and B2 (CD19+CD11b-, 3.9±1) subpopulations. Importantly, both α-m and γ-m2, that totally lack HS-binding activity, failed to attract leukocytes either at 6 hr or 15 hr time points.

**Figure 7:**
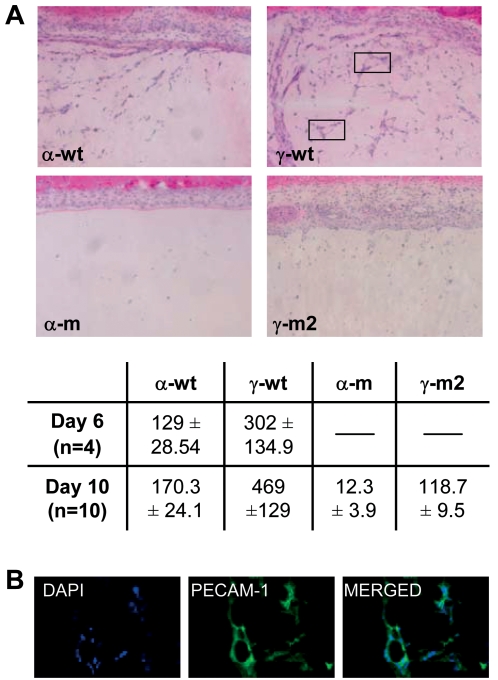
Angiogenic effect of α-wt or γ-wt and derivatives chemokines. (A) Haematoxylin-eosin staining of Matrigel plugs containing 10 nM of the indicated chemokine and analyzed at day 10 from implantation in BALB/c mice. Data are representative of ten independent experiments. In frame, vessel-like structures forming arround a central lumen. In the table, quantification of the number of migrated cells (±SD) into the Matrigel after 6 (p = 0.0459) or ten days (p = 0.0009) from implantation. (B) Immunofluorescent detection of PECAM-1/CD31^+^ endothelial cells in Matrigel neovessels with DAPI nuclear counter-labelling. Original magnifications ×100 (A) and ×600 (B).

CXCL12α has the capacity to promote *de novo* formation of vessels, a property related to the ability of this chemokine to regulate both the traffic and survival of stem and progenitor cells [19], [35]. Thus, we compared the ability of γ-wt and α-wt to attract endothelial progenitors and initiate the angiogenic process. To this purpose, Matrigel plugs loaded with an endotoxin-free, 10 nM solution of either γ-wt or α-wt were implanted subcutaneously in BALB/c mice. Whereas virtually no infiltrating cells were detectable in control PBS Matrigel plugs (data not shown), γ-wt induced a more robust response (3-fold increase, p = 0.0009 Figure 7A) than α-wt regarding the total number of cells attracted at day 10 post-implantation. Vessel-like cellular tubes within Matrigel implants were particularly abundant in γ-wt-loaded implants. These vessel-like structures were mainly composed of endothelial cells expressing CD31/Platelet endothelial cell adhesion molecule (PECAM-1) (Figure 7B), a molecule that defines endothelial cells. Similar results were observed 6 days post-implantation, the minimal time-point required to observe angiogenesis using this technique [36] (p = 0.0459, Figure 7A table). Of note, both α-m and γ-m2 display a reduced capacity to promote cell infiltration and angiogenesis in Matrigel implants, demonstrating the importance of GAG binding for this process.

## Discussion

*Cxcl12γ* mRNA has been primarily detected in the central nervous system of adult rats, where this isoform is supposed to undergo inverse regulation as compared to the β isoform both during brain development and in pathophysiological events like sciatic nerve lesion [26]. *Cxcl12γ* mRNA is also differentially expressed in normal and myocardial infarcted rat heart [37], in normal and ischemic brain of mice [38], and is broadly detected in human adult tissues [27]. Here, we have characterized for the first time the expression of CXCL12γ at the protein level. The apparent exclusion of both *Cxcl12γ* mRNA and protein from discrete places suggests that the expression of this isoform is tightly regulated by a RNA-splicing regulatory mechanism. Remarkably, CXCL12γ seems to be expressed in anatomical sites, such as small vessels and lower respiratory tract, where it could be involved in the diapedesis of inflammatory leukocytes and other cells from hematopoietic origin. In embryo, its enhanced capacity to form haptotactic gradients could be critical for guiding discrete cell precursors into their final localization during organogenesis.

The tight array of BBXB motifs in the CXCL12γ C-ter domain, that distinguish this protein from other CXCL12 isoforms, is unprecedented among HS-binding proteins. The C-ter domain has on its own a marked affinity for heparin that decreases dramatically when HS-binding motifs are mutated. This observation is in keeping with our results issued from a Nuclear Magnetic Resonance analysis of the soluble form of this chemokine [28], which revealed that the C-ter peptide is unfolded and could offer an accessible, highly cationic surface for the molecular recognition in the interaction with GAG. Our interpretation of SPR findings is that the high affinity for the oligosaccharide displayed by γ-wt largely relies in the low k_off_ of the HP/γ-wt complexes which has been estimated to be 0.0019 M^−1^ s^−1^, contrasting with the rapid dissociation from HP observed for α-wt (k_off_ 0.111 M^−1^ s^−1^)[28]. This is well exemplified by the SPR profile obtained with the mutant γ-m1. This mutant dissociates more rapidly from HP and shows a marked, reduced interaction with HP as compared to the wild type counterpart. However, it retains a substantial affinity for HP that might result from the stabilization of the complex through the collaboration between the conserved BBXB motif in the core of the chemokine and the remaining positive charges in the yet highly cationic C-ter domain. Collectively, these data underline the important contribution of the C-ter BBXB motifs to the formation of high-affinity and stable HP/γ-wt complexes.

The chemokine-binding experiments carried out in intact cells proved the specificity and high affinity of γ-wt for cellular HS structures, thus validating the biological relevance of the *in vitro* analysis. The astounding strong interaction of γ-wt with cell GAG was also observed in an alternative assay. Indeed, our findings prove that γ-wt is massively adsorbed at the cell surface following secretion. The simplest explanation for this phenomenon is that the secreted γ-wt could be rapidly trapped on cell-surface HS structures. Alternatively, the high affinity of γ-wt for HS might result in the formation of an intracellular complex before being expressed at the cell surface, a phenomenon previously described for the Fibroblast Growth Factor-2 [39]. On view of the low dissociation rate of HS-γ-wt complexes, it can be speculated that the secreted, free form of the chemokine hardly would reach the equilibrium of interaction with immobilized HS and that under physiological conditions, the binding of natural CXCL12γ to extracellular HS structures is tight and long-lasting.

Using lymphoid T cells, we confirm that γ-wt signals through CXCR4 with diminished agonist potency as compared to α-wt. This can be accounted for by the decreased affinity of γ-wt for CXCR4 that was previously reported [28]. It can be hypothesized that, either the electrostatic interactions of the highly cationic C-ter domain with the negatively charged N-ter domain and extracellular loops of CXCR4 [40], or the steric hindrance promoted by the bulky basic residues in the γ-wt C-ter domain, impair the specific interaction with CXCR4 and therefore reduce the agonist potency of γ-wt.

Importantly, neutralization of positive charges in the BBXB motifs of γ-wt (γ-m1 and γ-m2) leads to an increased affinity for [28] and activation of CXCR4 comparable to this achieved either by α-wt or α-m, two proteins that preserves similar overstructure and efficiency towards CXCR4 [31]. Collectively, these findings conclusively identify the charged C-ter domain as responsible for the distinctive structural and cell-signaling properties showed by γ-wt. In contrast to the situation observed for CXCR4, γ-wt binding to CXCR7 is comparable to this of α-wt, suggesting that molecular determinants of CXCL12 for binding to CXCR4 and CXCR7 are different.

The demonstration of *in vivo* consequences of chemokine/GAG interactions have been hampered by conformational changes consecutive to the mutagenesis of BBXB consensus sites that leads frequently to an overall reduced affinity of the chemokine for the corresponding receptor [22]. The naturally occurring CXCL12γ protein is free of this bias and offers an unprecedented opportunity to ascertain the importance of chemokine/GAG complexing in the regulation of *in vivo* cell migration in adult life.

The capacity of endogenous CXCL12α to promote leukocyte attraction in the peritoneum has been proved previously [41]. Similarly, it has been demonstrated that the formation of vessels under physiological [19] and pathological conditions [35] is induced by CXCL12α and is related to the regulation of the traffic and survival of CD34+ progenitor cells. The secreted signalling protein Vascular Endothelial Growth Factor-A (VEGF-A) is another potent and specific angiogenic factor [42]. *Vegf-A* isoforms are produced by alternative splicing from a single gene [43] and their levels are exquisitely regulated through transcriptional control and mRNA stability [44]. Similarly to CXCL12, these isoforms differ by the absence or presence of protein domains that confer the ability to bind heparin and therefore could mediate their capacity to interact differentially with GAG components in the extracellular environment of VEGF-secreting cells [45]. In keeping with this assumption, it has been shown that the absence of the heparin-binding isoforms of VEGF-A alters the distribution and gradients of VEGF-A protein without heparin-binding capacity in tissues and leads to a reduced vascular branching complexity and increased microvessel calibre [46]. This situation is reminiscent of our observation showing that γ-wt displays an enhanced capacity to induce *in vivo* recruitment of cells as compared to α-wt, which is due to its enhanced affinity for HS. This conclusion is strongly supported by the fact that the HS-binding disabled mutant γ-m2 is devoid of detectable activity *in vivo* despite it shows full agonist potency on CXCR4 *in vitro*.

It has been shown that the association of HS with CXCL12α prevents the proteolytic attack of its N-ter domain by the endopeptidase CD26/DPP, thus preserving the functionality of the chemokine [23]. CXCL12γ encodes several serine-protease cleavage sites in its distinctive C-ter and is conceivable that, like for VEGF-A [47], the interaction of γ-wt with HS also protects this domain from proteolytic attack, thus contributing to the prolonged immobilization and increased half-life of CXCL12γ in tissues.

Overall, from our findings, we conclude that, despite its reduced agonist potency on CXCR4, both the prolonged immobilization and increased stability of γ-wt would determine the superior capacity of this isoform to promote chemotaxis *in vivo* as compared to α-wt.

The conserved structure and differential expression of CXCL12γ both during development and in homeostatic and pathological conditions in the adult, herald the important and specific role that it might play both in embryogenesis and in adult life. Its localization in vascular endothelia where leukocyte diapedesis occurs and pathogen host defense are initiated, suggests that this chemokine is key in the fine-tuning of immune responses. CXCL12γ would represent the paradigm of haptotactic proteins that critically promote the directional migration and tissue homing of cells and regulate important homeostatic and physiopathological functions.

## Materials and Methods

### Chemokine synthesis and monoclonal antibodies

Chemically synthesized peptides and chemokines were generated by the Merrifield solid-phase method, and evaluated for their purity and concentration by mass spectrometry and amino-acid hydrolysis, respectively, as described [31]. The endotoxin content of the protein stocks (100 µM) was determined using a highly sensitive Limulus test (Limulus Amebocyte Lysate kit, Cambrex). Recombinant, wild type (γ-wt_r_) or mutant (γ-m1_r_ and γ-K2427S_r_) CXCL12γ chemokines were also obtained from *E. coli* BL21 cells by using the pET17b expression vector, analysed by mass spectrometry and quantified by amino acids analysis as described [28]. The 6E9 mAb (IgG1κ) directed against the wild type CXCL12γ protein was generated by immunizing BALB/c mice with a linear peptide containing the last 30 amino-acids of the γ-wt mature isoform, as previously described [31]. The mAb clone K15C was generated against an N-ter peptide shared by all the CXCL12 isoforms [31].

### Heparin affinity chromatography and SPR-based binding assays

Heparin affinity chromatography of chemokines was performed as previously described [48] on a 1-ml Hitrap heparin column and submitted to gradient elution from 0.15 to 1 M NaCl in 20 mM Na_2_HPO_4_/NaH_2_PO_4_. For SPR experiments, size defined HP (6 kDa) was biotinylated at its reducing end and immobilized on a Biacore sensorchip as described [48]. For binding assays, 250 µl of chemokine solution (26, 39, 59, 89, 133 or 200 nM for α-wt and 3.3, 4.9, 7.4, 11, 16.6 and 25 for γ-wt, γ-m1 and γ-m2) was injected at a flow rate of 50 µl/min across control and HP surfaces, after which the formed complexes were washed with running buffer for 5 min. The sensorchip surface was regenerated with a 3 minutes pulse of 2 M NaCl. Control sensorgrams were subtracted on line from HP sensorgrams. Equilibrium data were extracted from the sensorgrams at the end of each injection and K_d_ were calculated using the Scatchard representation.

### Cloning of Cxcl12 isoforms

Brain tissue was obtained by dissection of a BALB/c adult mouse. Total RNA was obtained by using the Trizol reagent (Roche, Basel, Switzerland) and after phenol-chloroform purification, isopropanol precipitation and quantization, cDNA was synthesized using 1 µg of total RNA. *Cxcl12α*, *β* and *γ* cDNA sequences were amplified using the common forward primer 5′atattgcgcgcatggacgccaaggtcgtcgcc3′ and the isoform specific reverse primers 5′tacctggagaaagctttaaacaagtaagggcccaatat3′ for *Cxcl12α*, 5′gctttaaacaagaggctcaagatgtgagggcccaatat3′ for *Cxcl12β*, and 5′cgacagaagaagagaaaggctgcccagaaaaggaaaaactaggggcccaatat3′ for *Cxcl12γ*. Amplified sequences were subcloned in a pcDNA3.1 expression vector or in a plasmid containing the SFV genome deleted for structural genes (pSFV-1). For ease of detection, the sequence coding for the bovine rhodopsin C9-tag (TETSQVAPA) was added in frame at the 3′ end of the open reading frames (ORF) of the *Cxcl12α*- and *Cxcl12γ*-encoding constructs, giving rise to the α-wt C9 and γ-wt C9 proteins, respectively. Nucleotide substitution (serine-coding triplets) in the *Cxcl12γ*-C9 construct corresponding to K78/K80 or R86/K88 gave origin to the γ-C9^up^ and γ-C9^dw^ proteins, respectively.

### Expression of Cxcl12 isoforms

Production of defective SFV particles and infections were performed as described [49]. pcDNA3.1 constructs were transfected in HEK-293T cells by the calcium phosphate method. Culture supernatants from 18 hours (hr)-SFV infected or 48 hr-transfected cells were collected and cleared by centrifugation. For preparing cell lysates, cells were detached in PBS-EDTA, centrifuged and pellets were treated with lysis buffer (20 mM Tris pH 7.5, 100 mM (NH_4_)_2_ SO_4_, 10% Glycerol, 1× protease inhibitor and 1% Triton X-100) and thereafter, cleared by centrifugation. In some experiments, cells were washed for 5 minutes at 4°C with PBS or 1 M NaCl solution prior to cell lysis and centrifuged before collecting wash fluids.

### RT-PCR

Total cDNA from tissues was obtained as described above. The PCR reaction was carried out using the forward primer 5′tgcccttcagattgttgcac3′, common for all isoforms, and the isoform specific reverse primers 5′gctaactggttagggtaatac3′, 5′cttgagcctcttgtttaa3′, and 5′gctagcttacaaagcgccagagcagagcgcactgcg3′ for *Cxcl12α*, *Cxcl12β* and *Cxcl12γ*, respectively. β-actin, amplified using the forward 5′acactgtgcccatctagcagggg3′ and reverse 5′atgatggagttgaaggtagtttcgtggat3′ primers, was used as a loading control.

Quantitative real time-PCR was performed in Mx3005Tm QPCR System with a MxPro QPCR Software 3.00 (Stratagene, La Jolla, CA) and SYBR Green detection system. Forward and reverse primer pairs, designed using online Primer3 software Primer3input (primer3 www. Cgi v 0.2), were 5′cttcatccccattctcctca3′ and 5′gactctgctctggtggaagg3′ for *Cxcl12α* and 5′ cgctttgtaactcgctcctc3′ and 5′ cagggccttatgaagcacat3′ for *Cxcl12γ*. No amplification was observed in PCR control reactions containing only water as the template. The Livak method was used to analyze the relative quantification RT-PCR data.

### Immunoblotting

For immunoblotting analysis, samples were separated on 4–12% SDS-PAGE (Bio-Rad, Hercules, CA), transferred to Filter type PDVF sequencing membrane (Millipore, Billerica, MA) and probed either with the anti-C9 (1D4), K15C or 6E9 mAb. A HRP-sheep anti-mouse Ig was used as secondary antibody (Amersham, Buckinghamshire, UK). Immunodetection was visualized using a Super Signal West Pico chemiluminescent substrate Kit (PIERCE, Rockford, IL) and a Fujifim LAS-1000 apparatus.

### Immunohistochemistry

Paraffin-embedded, mouse tissue sections were deparaffinized and rehydrated through graded alcohols, rinsed with PBS, blocked with a TENG-T solution, and incubated overnight at 4°C with primary K15C, 6E9 mAb or buffer alone (control). Sections were then washed and incubated with an anti-IgG mouse alkaline phosphatase (1/200) for 1.5 hr. Immunostaining was revealed using NBT/BCIP as substrate. Human tissue immunostaining was revealed with an anti-IgG mouse biotinylated antibody and avidin-peroxidase system. Images were acquired using a Nikon Eclipse 80i microscope and a Nikon Digital Sight DS-L camera and software.

### Immunofluorescence detection of CXCL12γ

HEK-293T cells were transfected by the calcium phosphate method with 500 ng of the corresponding pcDNA3.1 expression construct, using the pcDNA3.1 insertless vector as a control and thereafter, cells were spread on polylysine-treated coverslips. Immunofluorescence was performed 48 hr after transfection. To promote intracellular accumulation of CXCL12, cells were treated with Brefeldin A (10 µg/ml) for the last 4 hr of culture. Cells were washed and fixed with 3.7% paraformaldehyde in PBS, permeabilized with PBS 0.2% BSA, 0.05% Saponine buffer for 30 min at 4°C, incubated with the 6E9 mAb for 30 min at 4°C, and finally incubated with Texas Red anti-mouse IgG secondary antibody (Vector Laboratories, Burlingame, CA). Images were taken using an inverted microscope Zeiss Axiovert 200 M piloted by Zeiss Axiovision 4.4 software and acquired with a CCD camera Roper Scientific Coolsnap HQ and analysed using the AxioVision LE program.

### Flow cytometry analysis

#### CXCL12 binding to cells

Adherent HEK-293T cells, Human Microvascular Endothelial Cells (HMVEC), parental CHO-K1 cells, HS-deficient CHO-pgsD677 or GAG-deficient CHO-pgsA745 mutant cells were incubated with the indicated concentration of chemokine for 60 min at 4°C. Unbound chemokine was removed by washing and cell-bound chemokine was detected with the K15C mAb. After washing, cells were stained with a PE-goat anti-mouse Ig secondary antibody (BD Pharmingen, San Jose, CA), fixed in 3% formaldehyde buffer and analyzed by flow cytometry (FACSscan, Becton Dickinson, CA). Statistical analyses of the differences in binding between the wild type proteins and their mutant counterparts were conducted using the Prism 5.0 software by fitting an unpaired two-tailed Student's t test.

#### CXCL12γ-C9 and CXCL12α-C9 distribution

Adherent HEK-293T cells were transfected with the corresponding pCDNA3.1 construct by the calcium phosphate method and cultured for 48 hr. Four hours before collecting them, the cell supernatants were removed and, when indicated, Brefeldin A was added to the fresh medium. Collected cells were left untreated or permeabilised with saponin and immunolabelled with the 1D4 mAb and a PE-goat anti-mouse Ig secondary antibody and analysed by flow cytometry. CXCR4 detection was performed using the PE-mouse anti-human CD184 (clone 12G5, all from BD Bioscience).

### Enzyme-linked immunosorbent assay

Quantification of chemokines was carried out using the DuoSet ELISA Development kit for mouse CXCL12 (R&D Systems, MN, USA). Statistical analyses were conducted using the Prism 5.0 software by fitting an unpaired two-tailed Student's t test.

### Functional assays

For G-protein activation assay, preparation of crude membrane fractions and [^35^S]GTγS binding were performed as described [50]. Data were analysed using non-linear regression applied to a sigmoidal dose-response model (variable slope) with the Prism 5.0 program (GraphPad Software Inc., San Diego, CA). For chemotaxis, freshly isolated CD4+ cells were obtained as described previously [51]. Migration of the lymphoblastoid cell line A3.01 or human primary CD4+ cells in response to CXCL12 was evaluated using a transwell system as described [52].

### Intraperitoneal recruitment assay

Two-month-old female BALB/c mice were intraperitoneally injected either with PBS alone (control) or the corresponding chemokine diluted in 300 µl of PBS at a concentration of 30 nM. Cells were recovered by washing the peritoneum with 20 ml of sterile PBS. Total number of cells per mouse was determined by trypan blue exclusion and they were phenotyped by flow cytometry analysis using the mAbs FITC-rat anti-mouse Gr-1, FITC-hamster anti-mouse CD3, PE-rat anti-mouse CD11b or APC-rat anti-mouse CD19 (all from BD Biosciences). Statistical analyses and model fitting of the effect of the wild type proteins and their mutant counterparts were conducted using the Prism 5.0 software by fitting an unpaired two-tailed Student's t test.

### Angiogenesis assay

Mouse subcutaneous Matrigel implants (BD Biosciences) were used as described [53]. Briefly, 500 µl of Matrigel containing 10 nM concentration of chemokines were subcutaneously injected in the back skin of female 2-mo-old BALB/c mice. The major component of Matrigel is laminin, followed by collagen IV, heparan sulfate proteoglycans, and entactin [54]. After 6 or 10 days, skin containing Matrigel plugs were excised. Frozen sections were fixed in 4% paraformaldehyde and analysed by haematoxylin-eosin staining or immunofluorescent labeling with an anti-CD31/PECAM-1 mAb (Santa Cruz, Ca, USA). Quantitative data were obtained by counting the number of cells (DAPI positive nuclei) per Matrigel area in digitalised images using ImageJ software (http://rsb.info.nih.gov/ij). Images were taken in a Zeiss Axioplan 2 microscope (Carl Zeiss, Jena , Germany) using a SpotRT CCD camera and Spot 4.5 software (Diagnostic Instruments, Sterling Heights , MI). Statistical analysis was conducted by fitting an unpaired two-tailed Student's t test, as described above.

## Supporting Information

Figure S1 Comparative GAG-binding activity of α-wt and γ-wt on parental CHO-K1 cells. Prior to incubation with the proteins, cells were treated with 10-3 units/mL of Heparinase (25°C), Heparitinase I (37°C) or Chondroitinase ABC (37°C) degrading enzymes (Seikagaku corporation, Tokyo, Japan) for 90 minutes. Cells were washed twice with PBS, detached with 2 mM EDTA in PBS and then assayed for CXCL12 binding as described previously (flow cytometry analysis). Binding to control untreated cells were arbitrary set to 100 and binding observed for enzyme-treated cells was expressed as a function of signal obtained in control conditions. Data are the mean±SD from three independent determinations. In inset, HS detection at the cell surface of control (K1) or Heparitinase I treated (K1+HT) CHO parental cells was performed using mouse IgM isotype control (gray-filled histogram) or the anti-HS mAb clone 10E4 and a PE-goat anti-mouse Ig secondary antibody.
(1.59 MB TIF)

Figure S2 CXCR7 is a high affinity receptor for both α-wt and γ-wt. (A) Specific CXCL12 α-CXCR7 interaction. 0.5 nM α-biot was added to A0.01 parental cells (Parental) or CXCR7-transduced A0.01 cells (CXCR7) and revealed by flow cytometry after addition of the streptavidin (SAv)-PE conjugate antibody (BD Bioscience) at 1 µg/ml. When indicated, α-biot binding to CXCR7 was inhibited using the mouse anti-human CXCR7 mAb (9C4, 50 µg/ml) or the α-wt chemokine (1 µM). Binding of SAv-PE alone to parental cells was arbitrary set to 1. (B) Concentration-dependent inhibition of 1 nM α-biot binding to CXCR7-transduced A0.01 cells by untagged α-wt or γ-wt chemokines. Cells were incubated with the indicated concentration of the chemokines, and after washing, labeled with 1 µg/ml of SAv-PE and analyzed by flow cytometry. Results are normalized for specific binding performed in the absence of competitor (100%, untreated). Binding parameters were determined with the Prism Software using non-linear regressions applied to one-site models. Results (mean±SD) are representative out of two (A) or three (B) independent experiments performed in duplicate.
(1.57 MB TIF)

Figure S3 AMD3100 effect in CXCL12-induced signalling. (A) [35S]GTPγS binding assay to membranes from lymphoblastoid A3.01 T cells upon activation with increasing concentrations of α-wt or γ-wt chemokines. When indicated 200 nM of AMD3100 was added to the incubation mix. Data are mean±SD of triplicate determinations from two independents experiments. (B) Chemotaxis of A3.01 cells. Chemokines were added to the lower chamber at a concentration of 3 nM for α-wt and 10 nM for γ-wt to obtain the maximal chemotactic effect for these cells. When indicated, AMD3100 was added to the upper and lower chamber at a final concentration of 200 nM. Results (mean±SD) are from two independent experiments and are expressed as percentage of input cells that migrated to the lower chamber.
(1.08 MB DOC)

Figure S4 Chemotactic activities of CXCL12 isoforms in activated leukocytes. (A) Primary lymphocytes blasted with phytohemagglutinin and expanded with IL-2 were left untreated or treated with Heparitinase I+Chondroitinase ABC (HT+CH) and incubated with the indicated concentrations of chemokine for 60 min at 4{degree sign}C. After extensive washing to remove unbound chemokine, cells were labelled with the K15C mAb and a PE-goat anti-mouse Ig secondary antibody. Fixed cells were analyzed by flow cytometry. Values represent the mean fluorescence intensity±SD of three independent experiments performed in triplicate. (B) Dose-dependent α-wt- or γ-wt-induced chemotaxis assessed in untreated and HT+CH-treated, activated primary lymphocytes. Results (mean±SD) are from two independent experiments and are expressed as percentage of input cells that migrated to the lower chamber.
(1.33 MB TIF)
