# Supplementary figures and images for: The CXCL12γ Chemokine Displays Unprecedented Structural and Functional Properties that Make It a Paradigm of Chemoattractant Proteins
Source: PLoS One. 2008 Jul 2;3(7):e2543. doi: 10.1371/journal.pone.0002543 (PMC2481281; doi:10.1371/journal.pone.0002543)

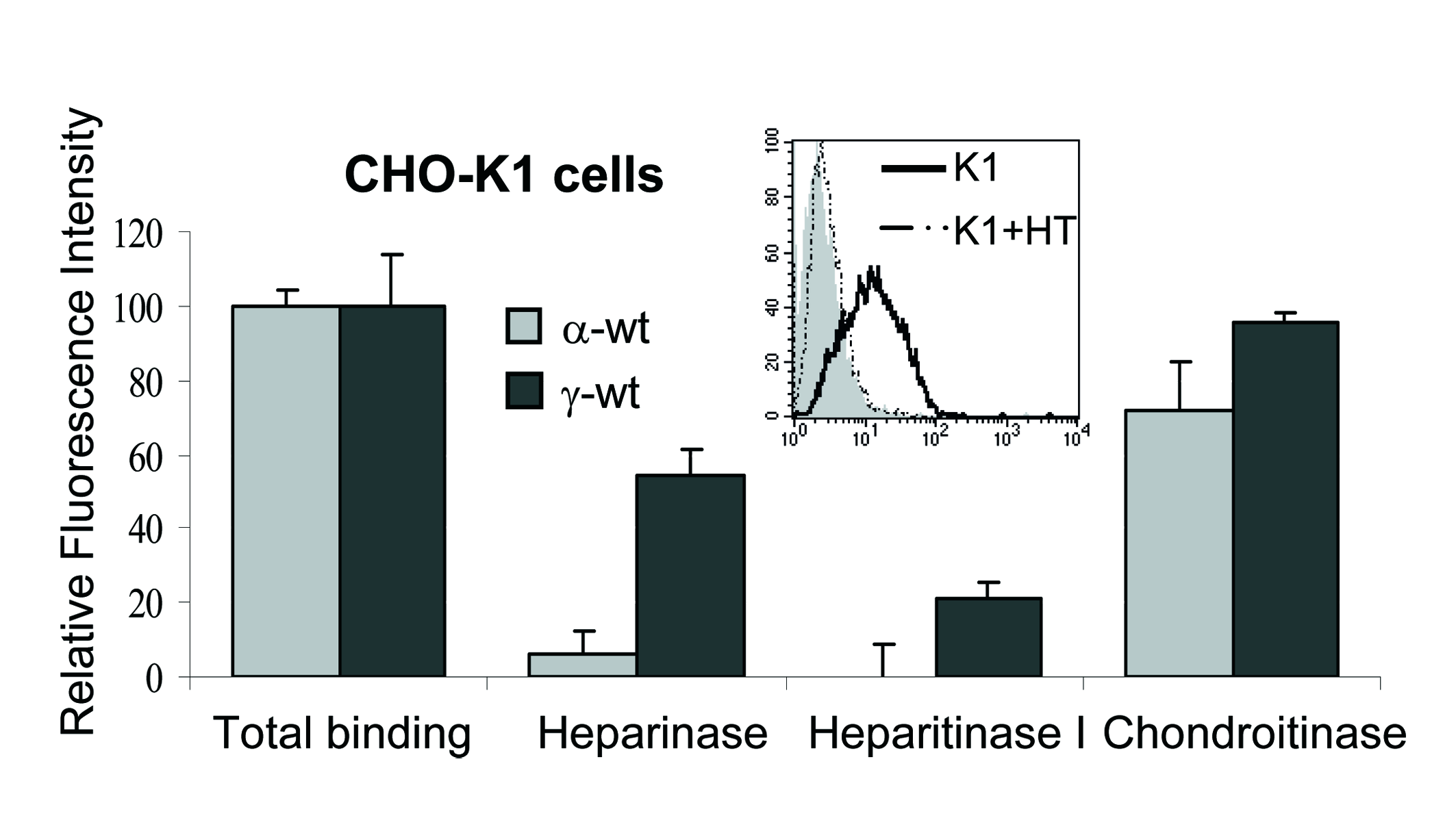

Supplement: Figure S1 — Comparative GAG-binding activity of α-wt and γ-wt on parental CHO-K1 cells. Prior to incubation with the proteins, cells were treated with 10-3 units/mL of Heparinase (25°C), Heparitinase I (37°C) or Chondroitinase ABC (37°C) degrading enzymes (Seikagaku corporation, Tokyo, Japan) for 90 minutes. Cells were washed twice with PBS, detached with 2 mM EDTA in PBS and then assayed for CXCL12 binding as described previously (flow cytometry analysis). Binding to control untreated cells were arbitrary set to 100 and binding observed for enzyme-treated cells was expressed as a function of signal obtained in control conditions. Data are the mean±SD from three independent determinations. In inset, HS detection at the cell surface of control (K1) or Heparitinase I treated (K1+HT) CHO parental cells was performed using mouse IgM isotype control (gray-filled histogram) or the anti-HS mAb clone 10E4 and a PE-goat anti-mouse Ig secondary antibody. (1.59 MB TIF) [file pone.0002543.s001.tif]

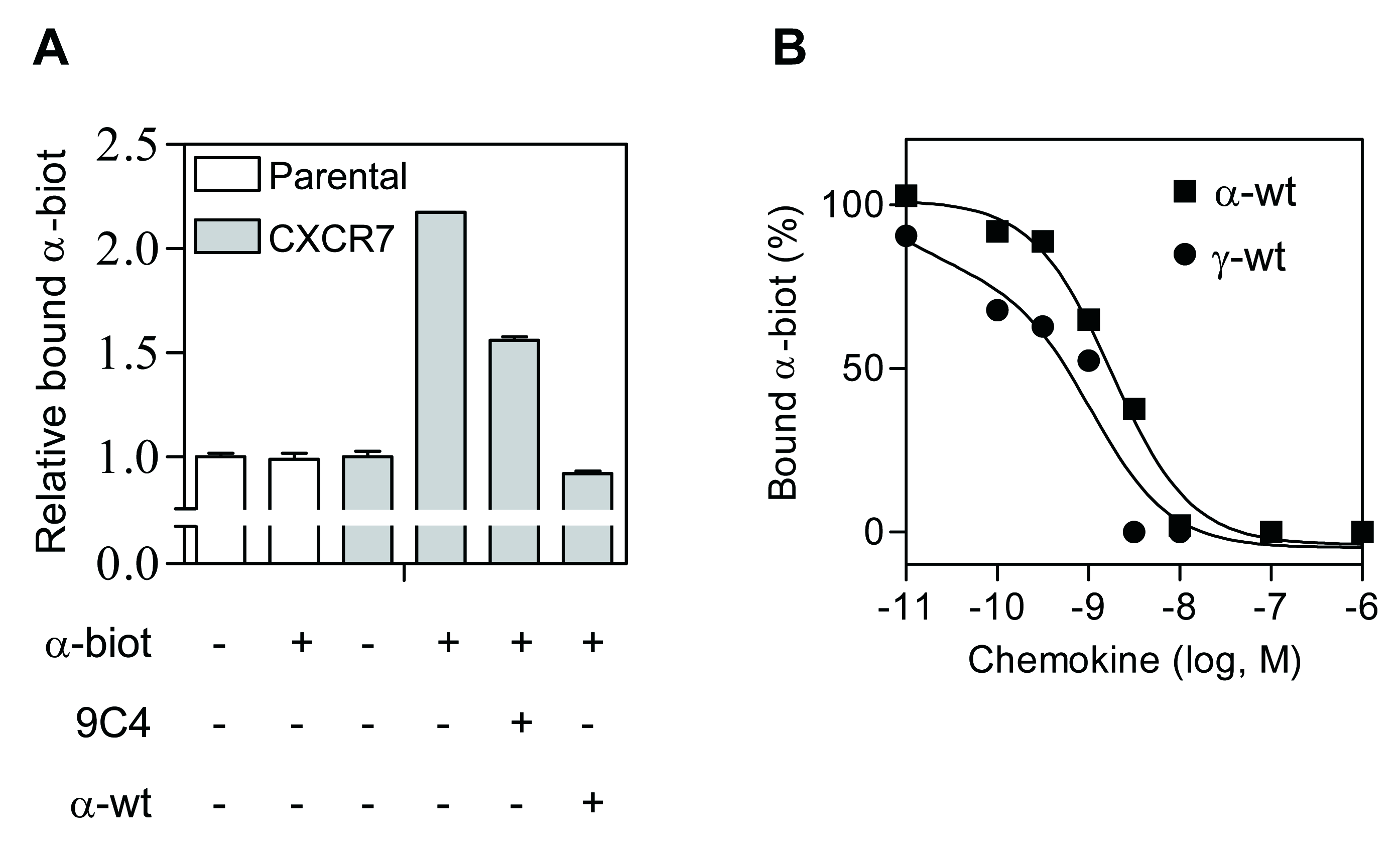

Supplement: Figure S2 — CXCR7 is a high affinity receptor for both α-wt and γ-wt. (A) Specific CXCL12 α-CXCR7 interaction. 0.5 nM α-biot was added to A0.01 parental cells (Parental) or CXCR7-transduced A0.01 cells (CXCR7) and revealed by flow cytometry after addition of the streptavidin (SAv)-PE conjugate antibody (BD Bioscience) at 1 µg/ml. When indicated, α-biot binding to CXCR7 was inhibited using the mouse anti-human CXCR7 mAb (9C4, 50 µg/ml) or the α-wt chemokine (1 µM). Binding of SAv-PE alone to parental cells was arbitrary set to 1. (B) Concentration-dependent inhibition of 1 nM α-biot binding to CXCR7-transduced A0.01 cells by untagged α-wt or γ-wt chemokines. Cells were incubated with the indicated concentration of the chemokines, and after washing, labeled with 1 µg/ml of SAv-PE and analyzed by flow cytometry. Results are normalized for specific binding performed in the absence of competitor (100%, untreated). Binding parameters were determined with the Prism Software using non-linear regressions applied to one-site models. Results (mean±SD) are representative out of two (A) or three (B) independent experiments performed in duplicate. (1.57 MB TIF) [file pone.0002543.s002.tif]

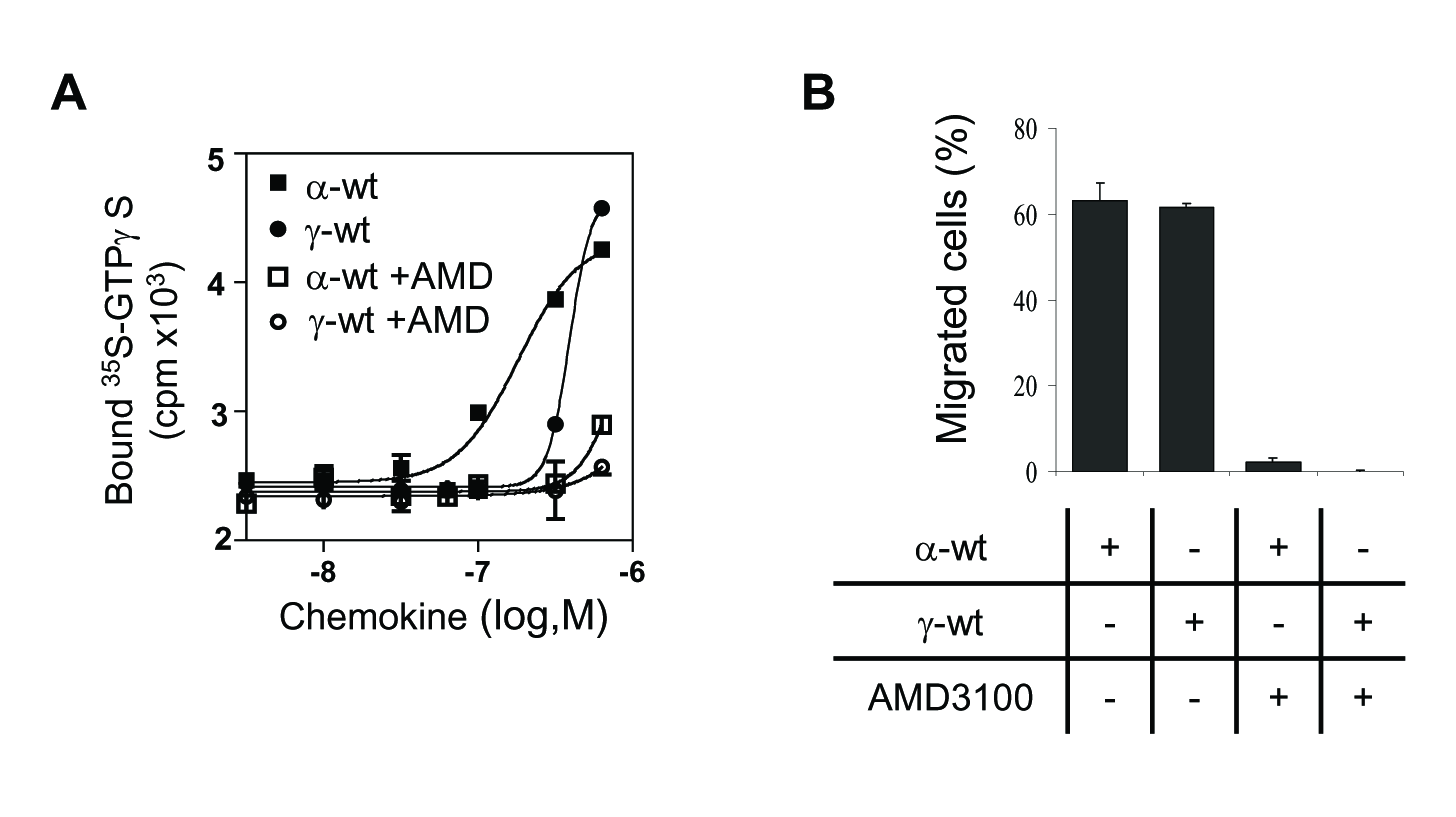

Supplement: Figure S3 — AMD3100 effect in CXCL12-induced signalling. (A) [35S]GTPγS binding assay to membranes from lymphoblastoid A3.01 T cells upon activation with increasing concentrations of α-wt or γ-wt chemokines. When indicated 200 nM of AMD3100 was added to the incubation mix. Data are mean±SD of triplicate determinations from two independents experiments. (B) Chemotaxis of A3.01 cells. Chemokines were added to the lower chamber at a concentration of 3 nM for α-wt and 10 nM for γ-wt to obtain the maximal chemotactic effect for these cells. When indicated, AMD3100 was added to the upper and lower chamber at a final concentration of 200 nM. Results (mean±SD) are from two independent experiments and are expressed as percentage of input cells that migrated to the lower chamber. (1.08 MB DOC) [file pone.0002543.s003.tif]

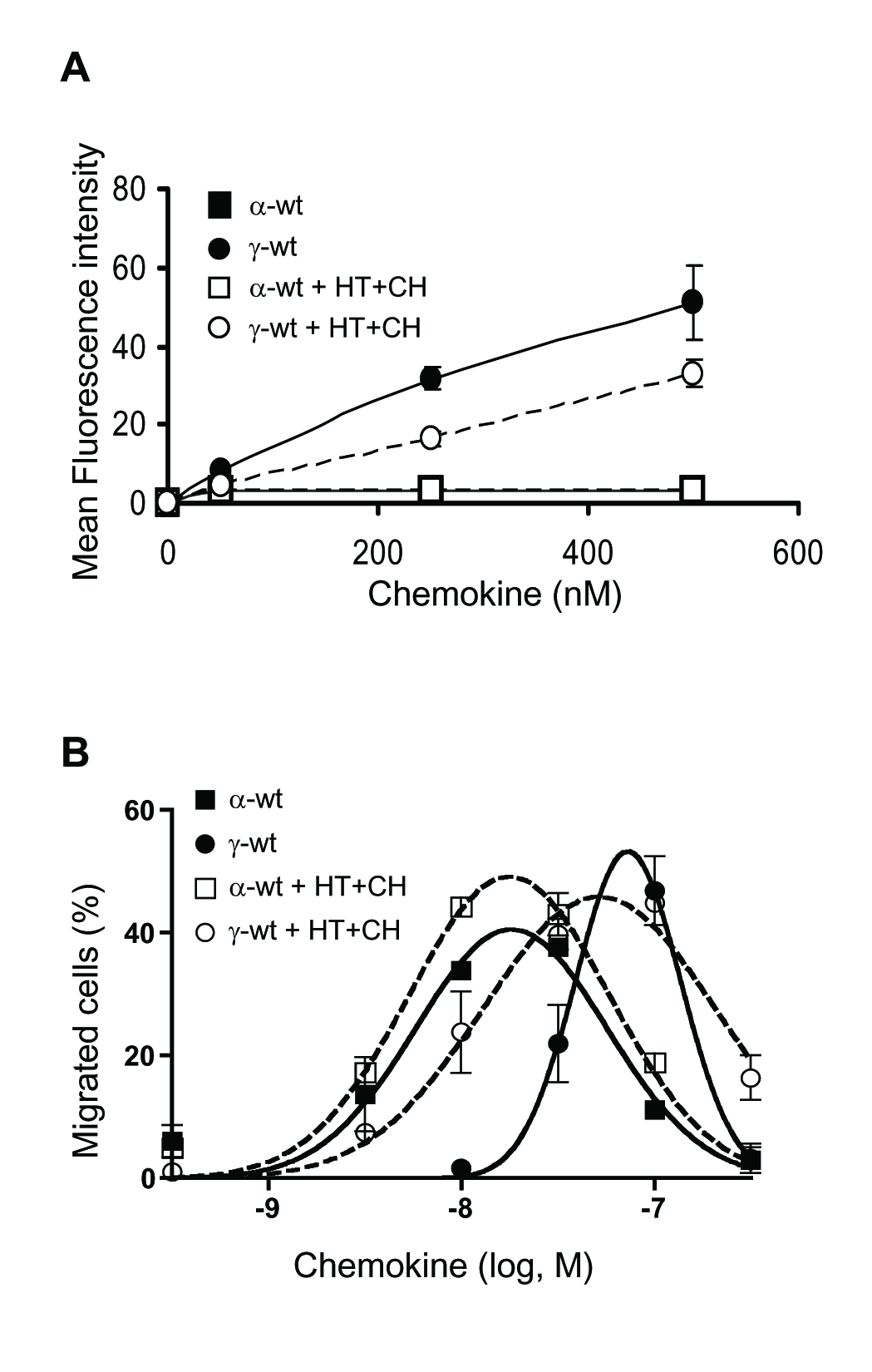

Supplement: Figure S4 — Chemotactic activities of CXCL12 isoforms in activated leukocytes. (A) Primary lymphocytes blasted with phytohemagglutinin and expanded with IL-2 were left untreated or treated with Heparitinase I+Chondroitinase ABC (HT+CH) and incubated with the indicated concentrations of chemokine for 60 min at 4{degree sign}C. After extensive washing to remove unbound chemokine, cells were labelled with the K15C mAb and a PE-goat anti-mouse Ig secondary antibody. Fixed cells were analyzed by flow cytometry. Values represent the mean fluorescence intensity±SD of three independent experiments performed in triplicate. (B) Dose-dependent α-wt- or γ-wt-induced chemotaxis assessed in untreated and HT+CH-treated, activated primary lymphocytes. Results (mean±SD) are from two independent experiments and are expressed as percentage of input cells that migrated to the lower chamber. (1.33 MB TIF) [file pone.0002543.s004.tif]
